# MEMOSys 2.0: an update of the bioinformatics database for genome-scale models and genomic data

**DOI:** 10.1093/database/bau004

**Published:** 2014-02-14

**Authors:** Stephan Pabinger, Rene Snajder, Timo Hardiman, Michaela Willi, Andreas Dander, Zlatko Trajanoski

**Affiliations:** ^1^Division for Bioinformatics, Innsbruck Medical University, 6020 Innsbruck, Austria, ^2^Health & Environment Department, AIT–Austrian Institute of Technology, Molecular Diagnostics, 1190 Vienna, Austria, ^3^Oncotyrol, Center for Personalized Cancer Medicine, 6020 Innsbruck, Austria and ^4^Development Anti-Infectives Microbiology, Sandoz GmbH, 6250 Kundl, Austria

## Abstract

The MEtabolic MOdel research and development System (MEMOSys) is a versatile database for the management, storage and development of genome-scale models (GEMs). Since its initial release, the database has undergone major improvements, and the new version introduces several new features. First, the novel concept of derived models allows users to create model hierarchies that automatically propagate modifications along their order. Second, all stored components can now be easily enhanced with additional annotations that can be directly extracted from a supplied Systems Biology Markup Language (SBML) file. Third, the web application has been substantially revised and now features new query mechanisms, an easy search system for reactions and new link-out services to publicly available databases. Fourth, the updated database now contains 20 publicly available models, which can be easily exported into standardized formats for further analysis. Fifth, MEMOSys 2.0 is now also available as a fully configured virtual image and can be found online at http://www.icbi.at/memosys and http://memoys.i-med.ac.at.

**Database URL**: http://memosys.i-med.ac.at

## Introduction

The extensive use of next-generation sequencing (NGS) technology in biological research has spurred the creation of genome-scale metabolic models for a multitude of organisms (1). Recently, new versions of metabolic reconstruction for the model organism yeast (2) and the human organism (3) were published and widely discussed in the research community.

Genome-scale models (GEMs) can be basically described as a network of metabolites that are connected by reactions occurring in the living organism. GEMs integrate various omics data types (bibliomics, metabolomics, proteomics) in a structured format, and can be used to perform computational and quantitative queries to answer various questions about the capabilities of the investigated organisms. Applications for GEMs are manifold, including biological discovery (4), flux analysis (5), gene deletion studies (6) and metabolic engineering (7). Furthermore, they can be used to provide an alternative context from which experimental data can be interpreted (4).

Over the past years, bacteria, fungi, as well as other organisms have been engineered to increase the yield of industrial enzymes and vitamins, as they are ideal hosts for the production of recombinant proteins. To further explore the characteristics of these important industrial microorganisms, GEMs have become more and more relevant for biotechnology to get a comprehensive understanding of its metabolism (8). The rapid development of methods for obtaining high-throughput data has now made it possible to study human metabolism on a genome-wide scale. GEMs provide a useful way to investigate numerous omics data types and address various research questions. Recently, human metabolic models have been used to study cancer metabolism (9) and predict potential drug targets and biomarkers (10).

The generation of new models is a well-defined iterative process comprising a multitude of different steps (11) where usually several intermediate revisions are generated (12). Owing to the vast amount of existing models, it is now reasonable to assume that new GEMs might be based on existing models of closely related organisms. During the construction process, the newly created GEM is compared with existing networks, and simulated results are constantly verified with experimental data. Therefore, it is of great importance to (i) have easy access to software for creating and manipulating models, (ii) be able to review all previous modifications, (iii) extract previous versions and (iv) export GEMs into a standardized format. Furthermore, the use of existing models for creating new GEMs might lead to bias where components of the novel organisms are underrepresented. Therefore, the ability to unequivocally compare models is of great help when designing new models.

To deal with these challenges, we previously developed the MEtabolic MOdel research and development System (MEMOSys) to support the construction, modification and management of GEMs (13). The web-based bioinformatics database stores all properties of a metabolic model and offers a powerful search system, a feature-rich comparison mechanism and standardized references to external databases. It uses a custom-version control system to automatically store the complete developmental history of all model components, which enables researchers to query and download all intermediate versions of a GEM. Furthermore, the database supports exchanging of models in the Systems Biology Markup Language (SBML) format.

Here, we report the second major release of MEMOSys, which introduces several updates to the database. The system has undergone a substantial redesign of the interface and business functionality and has been enhanced with a new feature to create derived models. Furthermore, new publicly available models were integrated into the database, and the complete MEMOSys system is now available as a fully configured virtual machine image. We believe that this major update to MEMOSys results in a sophisticated database that will facilitate the creation and investigation of GEMs by researchers worldwide.

## Results

The architecture and development platform of the database MEMOSys have been retained in this update as described in the original publication (13). However, the system has undergone significant changes to implement new functionality and features (the new database schema is depicted in Supplementary Figure S1). During the development of the database update, substantial effort has been devoted to testing the system and setting up a continuous integration test environment. Thereby, all functionality tests are executed for each added feature, which ensures that new functionality is compatible with the previous state.

An important cornerstone of MEMOSys is the compliance with the SBML. Import and export of models are now based on the native JAVA library JSBML (14), improving the overall performance of the system and considerably facilitating the installation of MEMOSys 2.0.

### Extension as a research database

As MEMOSys has proven to be a valuable research platform, we included several published GEMs into the database. The system now hosts 20 publicly available models (Table 1), which can be easily exported for further analysis. Original SBML models were modified and extended to be compatible with previously included model components. Among others, we have included the recently released models for *Saccharomyces cerevisiae* and *Homo sapiens*, as well as the model organisms *Mus musculus*, *Arabidopsis thaliana* and *Escherichia coli.*

**Table 1.** Listed are publicly available GEMs in the MEMOSys database

Model	Organism	Author	Reference
iLF583	*Amycolatopsis balhimycina*	Vongsangnak *et al.,* 2012	(15)
Arabidopsis	*Arabidopsis thaliana*	Mintz-Oron *et al.,* 2012	(16)
iHD666	*Aspergillus nidulans*	David *et al.,* 2008	(17)
iMA871	*Aspergillus niger*	Andersen *et al.,* 2008	(18)
iWV1314	*Aspergillus oryzae*	Vongsangnak *et al.,* 2008	(19)
iMZ1055	*Bacillus megaterium*	Zou *et al.,* 2013	(8)
iJO1366	*Escherichia coli*	Orth *et al.,* 2011	(20)
Recon1	*Homo sapiens*	Duarte *et al.,* 2006	(21)
Recon2	*Homo sapiens*	Thiele *et al.,* 2013	(3)
Llactis	*Lactococcus lactis*	Oliveira *et al.,* 2005	(22)
mmu_08	*Mus musculus*	Queck *et al.,* 2008	(23)
iAL1006	*Penicillium chrysogenum*	Agren *et al.,* 2013	(24)
iLC915	*Pichia pastoris*	Caspeta *et al.,* 2012	(25)
iSS884	*Pichia stipitis*	Caspeta *et al.,* 2012	(25)
iSB1139	*Pseudomonas fluorescens*	Borgos *et al.,* 2013	(26)
iFF708	*Saccharomyces cerevisiae*	Förster *et al.,* 2003	(27)
iIN800	*Saccharomyces cerevisiae*	Nookaew *et al.,* 2008	(28)
ymn2_0	*Saccharomyces cerevisiae*	Herrgard *et al.,* 2008	(29)
ymn7_0	*Saccharomyces cerevisiae*	Aung *et al.,* 2013	(2)
iIB711	*Streptomyces coelicolor*	Borodina *et al.,* 2005	(30)

### Derived model creation

**Figure 1.**
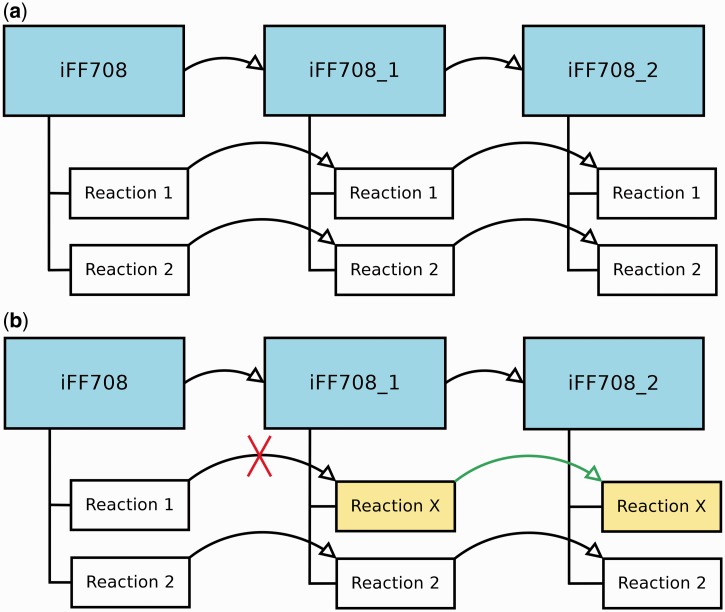
(**a**) Displays three GEMs where model iFF708_1 is a derived model of iFF708, and iFF708_2 is a derived model of iFF708_1. Model iFF708_1 and iFF708_2 are copies of model iFF708 and contain exact copies of all reactions present in model iFF708. (**b**) Shows a use case where Reaction 1 was modified in model iFF708_1 (its name was changed to *reaction X*) resulting in the loss of the reference to Reaction 1, and in the update of Reaction 1 in model iFF708_2. If Reaction 1 in model iFF708 is updated, reaction X will not be changed in model iFF708_1 and model iFF708_2.

During the construction and refinement of GEMs, it is often necessary to analyze models that deviate only in a few particular features. Therefore, MEMOSys 2.0 offers now the possibility to create derived models out of existing models without specifying a limit on the number of derived models. Each derived model is in essence an exact copy of the parent model, where the model itself and every reaction contain a reference to the parent. As soon as a property in the parent model is modified, MEMOSys implements a mechanism to propagate changes to all derived models. If a child reaction is modified, it loses its reference to the parent reaction and is now treated as an independent reaction. Figure 1 depicts the described scenarios.

**Figure 2.**
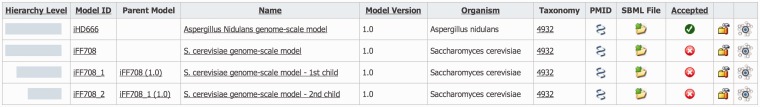
Depicted is the model list of MEMOSys. The first column graphically displays the hierarchical level of the model followed by various model properties. New derived models can be created by clicking on the icon displayed in the rightmost column.

Derived models are shown together with their parent model in the model list section of MEMOSys 2.0 (Figure 2). The system graphically depicts the hierarchical level of each derived model and lists the name of the respective parent model. To account for the changed data structure, the derived model history functionality was extended to include the modification history of the parent model. MEMOSys 2.0 is capable of listing all changes of a model, including its hierarchical dependencies. The model comparison mechanism, as introduced in the previous release, has been enhanced to allow the comparison of different derived models. Comparison result for reactions, metabolites and genes is either displayed in lists or graphically presented.

### Component annotation

**Figure 3.**
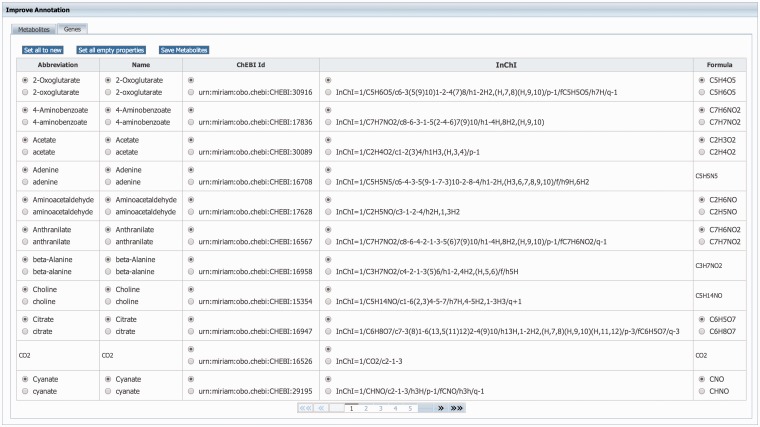
Displayed is the user interface for enhancing the annotation of metabolites and genes. After loading an SBML file, the system identifies new or different annotations and offers the possibility to select the correct annotation for each component. Furthermore, the user can choose to replace all current component annotations with the newly loaded ones, or store only new component annotations while keeping the currently existing annotations.

GEMs rely heavily on annotations to unambiguously identify model components. Therefore, MEMOSys supports annotating reactions, metabolites, genes and compartments with references to external databases using the ‘minimum information requested in the annotation of biochemical models’ (MIRIAM) (31) notation. To avoid redundancy and improve comparability and re-usability, MEMOSys stores only unique metabolites and genes in the database. As these components can be used by multiple reactions, updates or annotation changes are automatically visible for all attached reactions. To enhance these components with additional annotations, the newest version of MEMOSys allows users to load SBML files into the system to extract and store these annotations. Files are uploaded to the database using the file upload functionality. The parsing process can be started by dedicated users and after successfully loading the file, the system displays the annotations in a user-friendly way. Next, the user can select which annotations should be kept and stored in the database (Figure 3).

### Interface improvements

The user interface of MEMOSys 2.0 underwent a major overhaul. In general, unnecessary information was removed from the main pages, and frequently used features were put in more prominent places. The new version of MEMOSys provides a simple interface that allows users to directly browse to specific reactions using either its defined identifier or its KEGG ID. The improved model import mechanism displays several key figures (such as number of imported reactions, number of balanced/unbalanced reactions) after the process has finished providing the user with a quick overview of model characteristics. Another new feature allows enhancing reactions with citation information, where corresponding fields are automatically populated using valid PubMed IDs. The query mechanism for reactions was enhanced to enable searching for KEGG IDs, reactants and genes. To improve readability, the versioning for models has been redesigned, which is now consistently implemented throughout the whole database. Moreover, MEMOSys 2.0 offers a new BioCyc linking service using gene and organism IDs to create references to corresponding BioCyc Web sites. Based on the organism name, the system provides suggestions of matching IDs facilitating the assignment of correct organism IDs.

### Distribution

Two critical factors that contribute, among others, to the success of a bioinformatics software suite are easy installation (32) and the availability of a detailed user guide. Since its first release, the source code of MEMOSys has been open-source, and we provided step-by-step installation and usage instructions. The database can be installed on Linux and Mac, and since its version 2.0, also on Windows. Additionally, we now offer MEMOSys 2.0 as a ready-to-use virtual machine image including a fully configured system. The virtual machine image contains a PostgreSQL database, a JBoss application server, the MEMOSys system, as well as a web-based user-management application. Furthermore, the complete *Penicillium chrysogenum* model is already stored in the database, which contains numerous annotated components that can be used as a starting point for future GEM constructions.

## Discussion

Version 2.0 is a significant update to the MEMOSys database designed for the management, development and storage of metabolic models. The new derived model functionality allows researchers to build hierarchical relationships of models and quickly compare different versions of one GEM. Moreover, the provided propagation feature ensures that component updates are pushed to all derived models, which avoids inconsistency and cumbersome repetitive work.

It has been shown that the development of new models is facilitated by using previously inserted well-annotated components (11). Consequently, MEMOSys 2.0 includes a new annotation improvement functionality offering a simple way to add new annotations to already stored components. Owing to the flexible data design of MEMOSys, additional references to the existing databases can be easily added to all stored components. The inclusion of new models further increases the value of MEMOSys as a research system, where researchers can easily export and query one of the 20 existing GEMs. The authors of the database will continually improve the annotation of publicly available models, and new models will be included upon request.

In contrast to existing systems such as the Biochemical Genetic and Genomic (BiGG) database (33) or the BioModels database (34), MEMOSys support researchers during the reconstruction of novel metabolic models by providing an automatic auditing system. Furthermore, the database offers a rich web-based editing functionality for all components and features a comparison mechanism to quickly get an overview of model differences.

The distribution of MEMOSys 2.0 as a virtual machine will make it accessible to more users because it removes the burden of manual installation. Furthermore, the ability of virtual machines to reset states, save snapshots or export appliances presents tremendous opportunities for collaborative GEM development and model analysis. Additional analysis software can be installed directly into the virtual machine, and researchers can easily share the complete appliance including current and previous states of models by simply exporting it.

Since its initial release, the interface of MEMOSys has undergone several changes that addressed usability issues and design deficiencies. The improvements were based on long-term user feedback and include new query mechanisms, link-out services and page refinements. We believe that MEMOSys 2.0 will continue to be a useful tool for the research community, and the new update will facilitate the creation of new models and allow users to effectively explore and analyze existing GEMs.

## Supplementary Data

Supplementary data are available at *Database* Online.
